# Plasma membrane calcium ATPase 1 regulates human umbilical vein endothelial cell angiogenesis and viability

**DOI:** 10.1016/j.yjmcc.2021.03.011

**Published:** 2021-07

**Authors:** Alexandra Njegic, Agnieszka Swiderska, Charlotte Marris, Angel L. Armesilla, Elizabeth J. Cartwright

**Affiliations:** a Division of Cardiovascular Sciences, Manchester Academic Health Science Centre, Faculty Biology, Medicine and Health, University of Manchester, AV Hill Building, Oxford Road, Manchester M13 9PT, UK; b Centre for Tumour Microenvironment, Barts Cancer Institute, Queen Mary University of London, John Vane Science Centre, Charterhouse square, London EC1M 6BQ, UK; c Research Institute in Healthcare Science, Faculty of Science and Engineering, School of Pharmacy, University of Wolverhampton, Wolverhampton, WV1 1LY, UK

Angiogenesis encompasses a series of regulated processes enabling endothelial tip cell migration, stalk cell proliferation and eventual vessel anastomosis and maturation. Recently, a role for plasma membrane calcium ATPase (PMCA), specifically PMCA4, in negatively regulating angiogenesis has been identified *in vitro* and *in vivo*. Knockdown of PMCA4 in human umbilical vein endothelial cells (HUVECs) leads to an increase in tubule formation and upregulation of regulator of Calcineurin 1 (RCAN1.4) signaling, a downstream effector of the calcineurin/nuclear factor of T-cells (NFAT) pathway [1]. Furthermore, *in vivo*, functional suppression of PMCA4 leads to elevated reperfusion following induction of hindlimb ischaemia [1,2]. Given their function as Ca^2+^ extrusion pumps it is unsurprising that siRNA mediated knockdown of either PMCA1 or PMCA4 in HUVECs results in increased [Ca^2+^]_i_ [[3], [4], [5]] which has been shown to inversely correlate with endothelial sheet migration speed [3]. Recently, a mechanistic role for PMCA1 in endothelial nitric oxide synthase phosphorylation and insulin-dependent AKT activation has been described [4,5]. However, the implications of PMCA1 knockdown on angiogenesis has yet to be determined.

Knockdown of *ATP2B1* (si-PMCA1), *ATP2B4* (si-PMCA4), or non-targeting (si-NT) scrambled control (Dharmacon) in HUVECs (TCS Cellworks) was achieved using published methods [1,2]. To study wound migration, HUVECs were plated onto ImageLock 96-well plates (3 × 10^4^ cells per well) and the scratch wound generated using the Incucyte ‘woundmaker’ (Sartorius). Images were captured and analysed using the Incucyte S3 Live Cell Analysis system and Zoom® analysis centre respectively. To determine tubule formation, HUVECs were plated onto Geltrex™ Low Growth Factor Matrix (Invitrogen, 3 × 10^4^ cells per well) in Medium 200 (0.2% FBS) containing either PBS or 25 ng/ml VEGF_a-165_ (PeproTech). Following a 24-h incubation, wells were imaged using a Leica M165Fc stereomicroscope and LASX software (Leica). Tubules were quantified using Angiosys (version 2.0, TCS Cellworks). Western blot was performed on protein extracted from either basal or VEGF_a-165_-stimulated HUVECs using SDS-PAGE gel electrophoresis and Trans-Blot® Turbo™ (BioRad) protein transfer. Membranes were probed for the following: PMCA1, MCM7 (Santa-Cruz), MCM2, β-actin (Cell Signaling), MCM6 (ProteinTech), DSCR1 (Sigma) and α-tubulin, Na^+^/K^+^ ATPase (Abcam).

siRNA mediated *ATP2B1* knockdown significantly reduces the protein expression of PMCA1 (Fig. 1A) and leads to an increase in intracellular Ca^2+^ (data not shown). Transient PMCA1 knockdown results in decreased HUVEC viability over a period of days, altered cell cycle kinetics (Supplementary Fig. 1A and 1B) and significantly reduced expression of components of the key cell cycle mediator, the minichromome complex (MCM, Fig. 1C). Interestingly, depletion of MCM occurs exclusively upon PMCA1, and not PMCA4, depletion (Supplementary Figs. 1D—1F). Furthermore, PMCA1 knockdown leads to impaired HUVEC migration; quantification of wound density and wound width revealed both are significantly reduced in si-PMCA1 HUVECs (Fig. 1B). Impaired endothelial cell migration can impact on vessel formation and, when compared to VEGF_a-165_-stimulated si-NT controls, loss of PMCA1 results in a less complex *in vitro* vascular network, characterised by the formation of significantly fewer endothelial junctions and tubules (Fig. 1D). Lastly, the protein expression of RCAN1.4 is also upregulated in response to VEGF_a-165_-stimulation which occurs to a significantly greater extent in si-PMCA1 HUVECs when compared to controls (Fig. 1D).

**Fig. 1:**
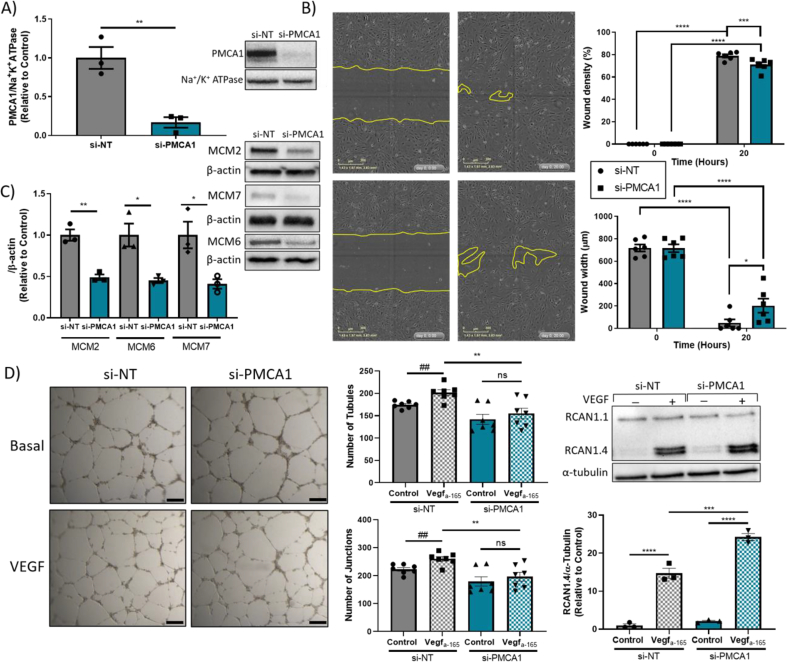
ATP2B1-silenced HUVECs show impaired wound migration, downregulated expression of minichromosome complex components 2, 6 and 7 and reduced tubule complexity. A) *ATP2B1*-targeting siRNA significantly depletes PMCA1 protein level (Students *t-test*, **p = 0.006, *n* = 3. B) IncuCyte real-time imaging of migrating HUVECs following generation of a scratch wound at Time 0- and 20-h post-scratch (scale bar = 300 μm). At Time20, wound density is significantly reduced and wound width significantly increased in si-PMCA1 HUVECs when compared to si-NT (Repeated measure two-way ANOVA with Sidak's multiple comparison, wound density ***p = 0.0008, ****p < 0.0001 and wound width *p = 0.0361, ****p < 0.0001, *n* = 6 independent experiments, with a minimum of 3 technical repeats per experiment). C) Representative Western blot images and densitometric analysis showing reduced PMCA1 expression leads to a significant downregulation of components of the putative helicase minichromosome complex (Students *t-test*, MCM2 **p = 0.0026, MCM6 *p = 0.0178, MCM7 *p = 0.0259, *n* = 3). D) Representative images of *in vitro* tubule formation following PMCA1 knockdown (scale bar = 250 μm). Quantification of the number of junctions and tubules shows inhibition of endothelial cell morphogenesis in si-PMCA1 HUVECs when compared to si-NT HUVECs under VEGF_a-165_-stimulated conditions (# Students *t-test,* number of junctions ^##^*p* = 0.0035, number of tubules ^##^*p* = 0.0024 and *two-way ANOVA, number of junctions **p = 0.071, number of tubules **p = 0.0055, *n* = 7 independent repeats with a minimum of 3 technical repeats). Representative Western blot image and subsequent densitometric analysis shows VEGF_a-165_ is a potent activator of RCAN1.4 expression in both groups; however, there is a significant increase in RCAN1.4 expression in si-PMCA1 when compared to non-targeting control levels HUVECS following VEGF_a-165_ –stimulation (two-way ANOVA, ***p = 0.0002, ****p < 0.0001, *n* = 3). All data shown are mean ± SEM, analysis performed using GraphPad Prism.

Here we demonstrate that loss of PMCA1 impairs basal endothelial cell migration and VEGF-mediated tubule formation *in vitro*; and leads to a reduction in core components of the putative DNA helicase MCM. These characteristics are in contrast to recently published data which demonstrated that transient PMCA4 knockdown increases both HUVEC migration and VEGF-dependent tubule formation [1], suggesting that PMCA1 and PMCA4 may play opposing roles in angiogenesis. Interestingly, both PMCA1 and PMCA4 knockdown models exhibit a VEGF_a-165_-dependent increase in RCAN1.4; however, in the context of PMCA1 knockdown, upregulation of RCAN1.4 may further impair tubule formation. Overexpression of RCAN1 supresses VEGF-mediated angiogenesis *in vitro* [6] and could therefore be inhibiting tubule formation in si-PMCA1 HUVECs. Furthermore, both PMCA1 and PMCA4 have been suggested to be putative regulators of VSMC growth and proliferation [7,8]. VSMCs isolated from PMCA4 knockout mice exhibit G1 cell cycle arrest [8], whereas it is an increase in PMCA1 in VSMC which causes a decrease in cell proliferation rate [7]. Conversely, in this study, it is a reduction in PMCA1 in HUVECs that reduces cell viability over time (Supplementary Fig. 1); interestingly, si-PMCA1 HUVECs exhibit a significant downregulation in MCM complex components basally. The MCM complex functions as a putative DNA helicase during DNA synthesis and its nuclear expression is tightly regulated to prevent re-replication [9]; however, MCM proteins exist in abundance and can function independently of their heterohexamer [10]. Furthermore, the expression of MCM2, MCM6 or MCM7 does not appear to be regulated by PMCA4 (Supplementary Fig. 1C—1F).

Taken together, there is a clear distinction between the role of PMCA1 and PMCA4 in angiogenesis *in vitro*; PMCA4 is a negative regulator, whereas PMCA1 positively regulates endothelial cell angiogenic phenotypes and viability.

## Funding

This work was supported by the 10.13039/501100000274British Heart Foundation [grant numbers FS/15/67/32038, FS/17/67/33483 and FS/19/60/34899] and The 10.13039/501100000770University of Manchester.

## Declaration of Competing Interest

None.

## Appendix A Supplementary data

Supplementary Fig. 1 Image 1
