# Supplementary material for: Plasma membrane calcium ATPase 1 regulates human umbilical vein endothelial cell angiogenesis and viability
Source: J Mol Cell Cardiol. 2021 Jul;156:79–81. doi: 10.1016/j.yjmcc.2021.03.011 (PMC8234512; doi:10.1016/j.yjmcc.2021.03.011)

***
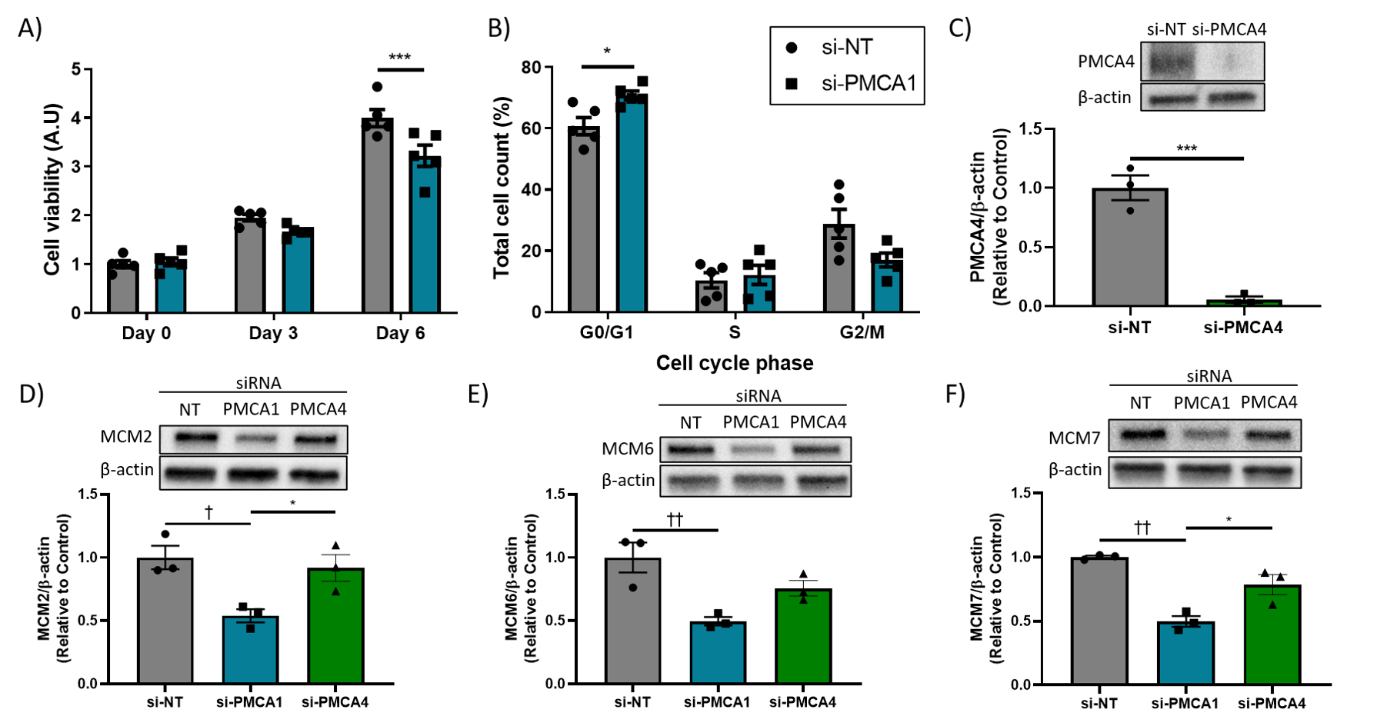
***

***Supplementary Figure 1: ATP2B1 depletion in HUVECs leads to a reduction in cell viability and altered cell cycle dynamics; characterised by a reduction in components of the minichromosome complex which occurs exclusively under PMCA1 and not PMCA4 knockdown conditions.***

A) The loss of PMCA1 leads to a reduction in HUVEC viability over time. Viability, assessed using Alamar Blue, was significantly decreased 6 days following completion of the siRNA transfection protocol (Repeated measure two-way ANOVA with Sidak’s multiple comparison test, ***p<0.0008, *n*=5, with a minimum of 3 technical repeats per experiment). B) Cell cycle dynamics assessed using FACs following a 72-hour period of serum starvation. Quantification of DNA content shows mild changes to the proportion of HUVECs in each phase of the cell cycle. *ATP2B1* depletion results in significantly more cells within G0/G1 phase of the endothelial cell cycle which corresponds with a lower proportion of cells within G2/M phase of the cell cycle (Students *t-test,* *p<0.013, *n*=5). C) siRNA mediated knockdown of ATP2B4 significantly reduces protein expression of PMCA4 (Students *t-test*, ***p<0.0009, *n* = 3). D, E and F) Knockdown of PMCA4 does not significantly alter the expression of MCM2, MCM6 or MCM7, suggesting the downregulation of MCM protein complex occurs exclusively upon *ATP2B1* depletion (One-way ANOVA, MCM2 †p=0.0217, *p=0.0482, MCM6 ††p=0.0099, MCM7 ††p=0.0011, *p<0.0183, *n*=3).

**Western blot images**

Figure 1A: Top: PMCA1, bottom: Na^+^/K^+^ ATPase housekeeper


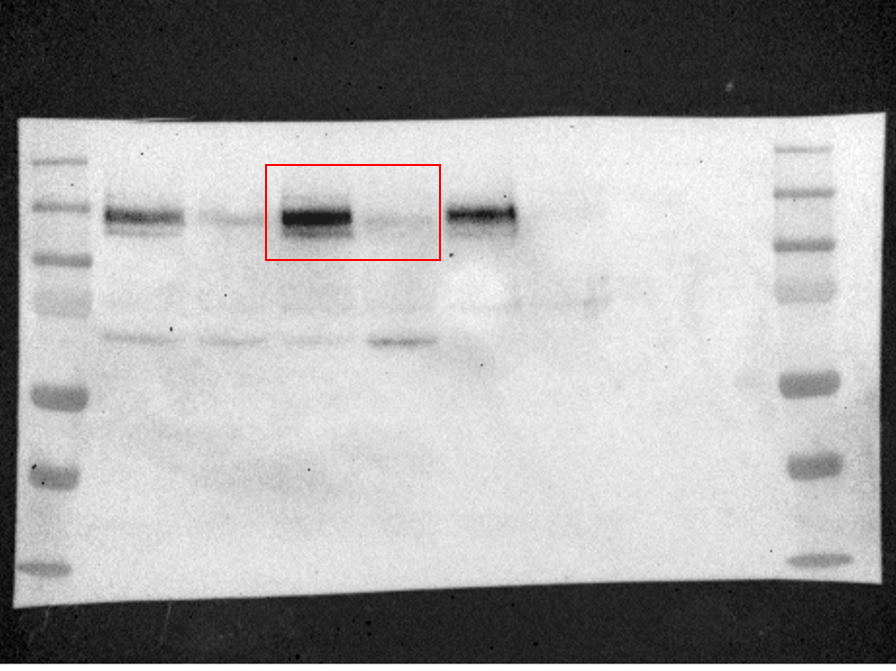

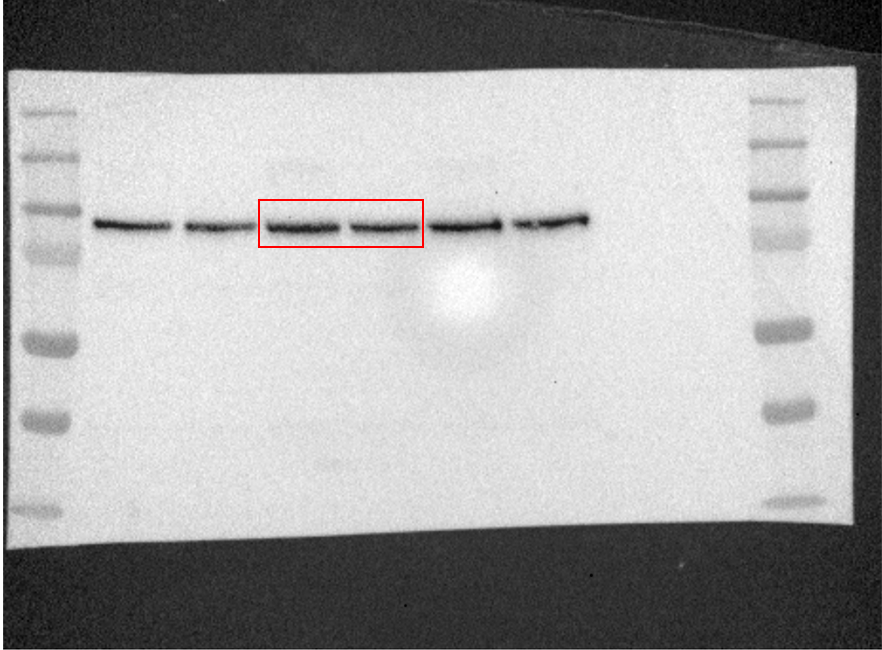


Figure 1C: Top: MCM2, bottom: β-actin


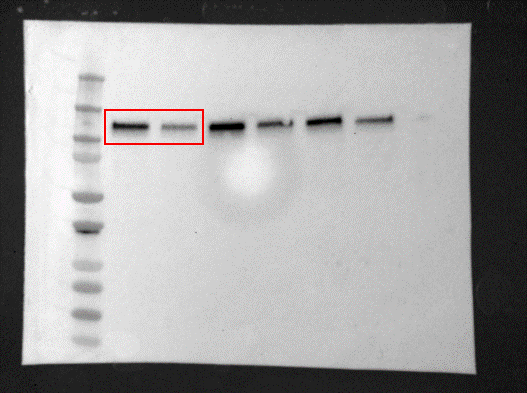

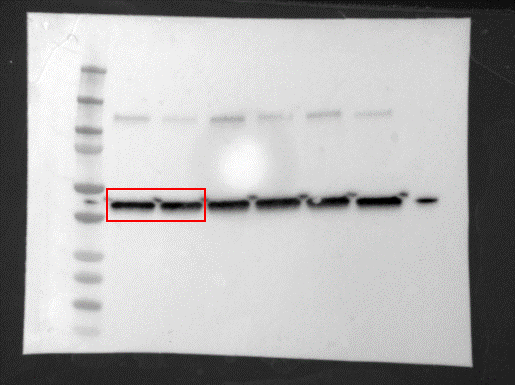


Figure 1C: Top: MCM6, bottom: β-actin


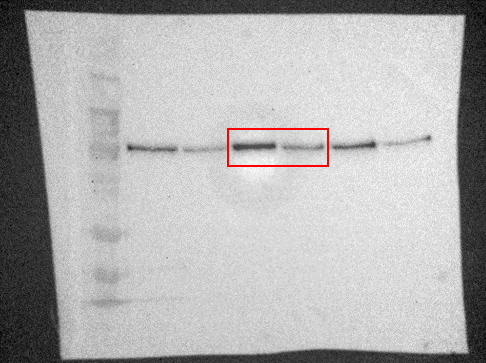

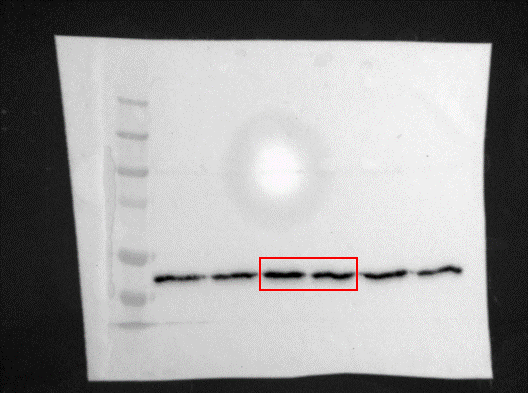


Figure 1C: Top: MCM7, bottom: β-actin


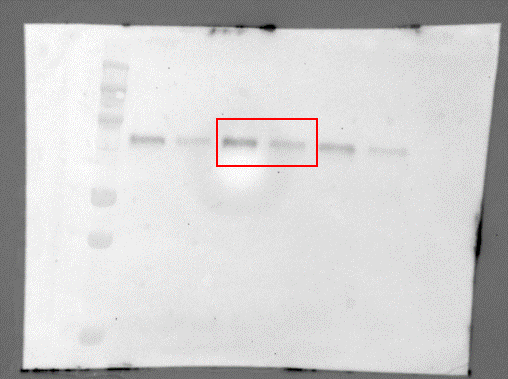

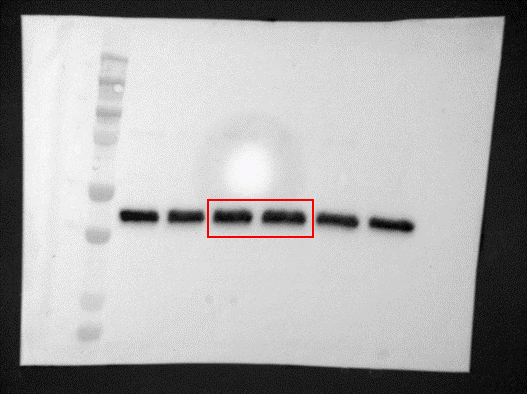


Figure 1D: Top: RCAN1.1 and RCAN1.4, bottom: α-tubulin housekeeper


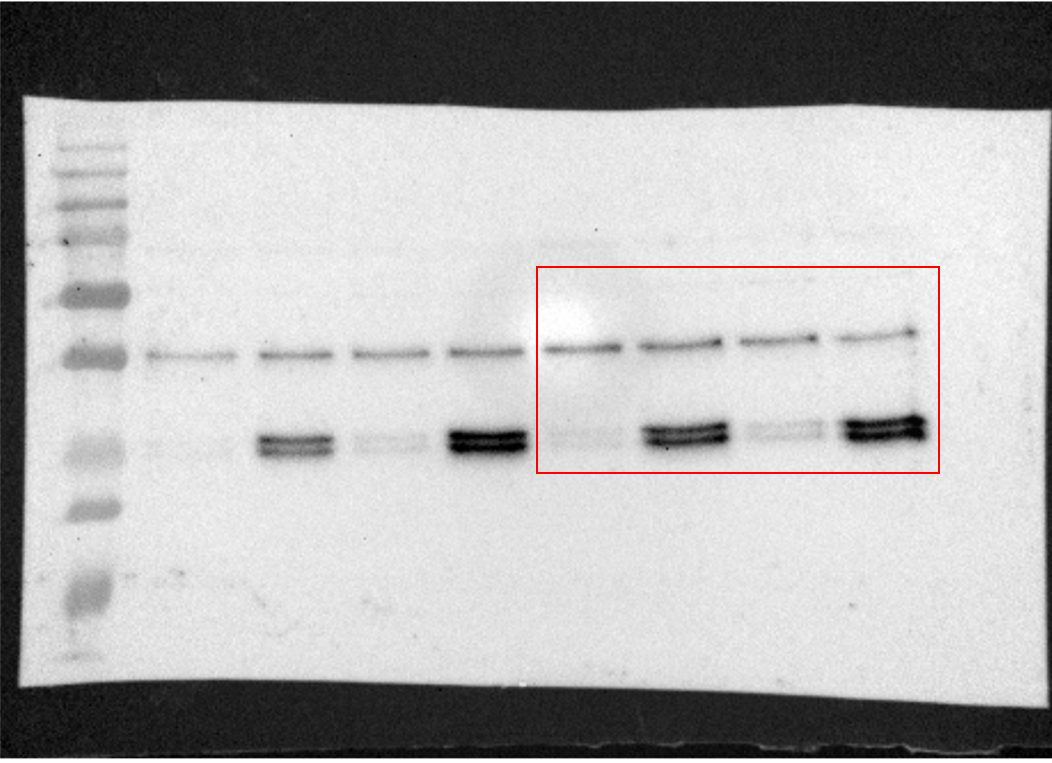


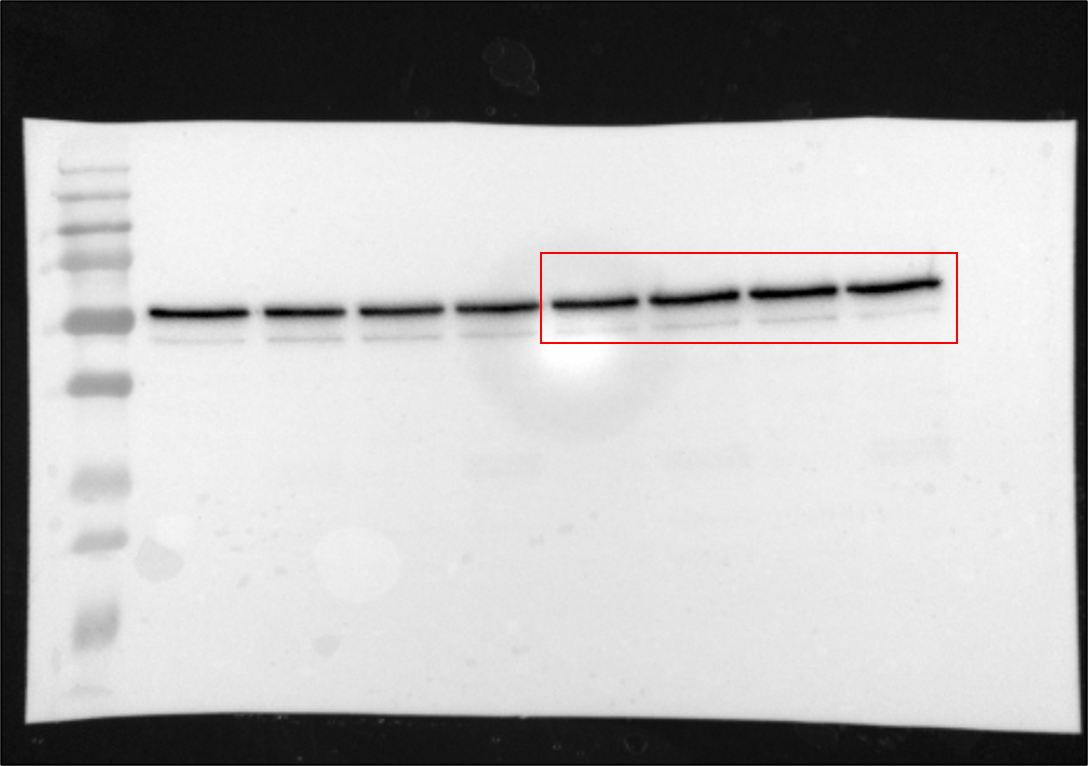


Supplementary Figure 1C: Top: PMCA4, bottom: β-actin


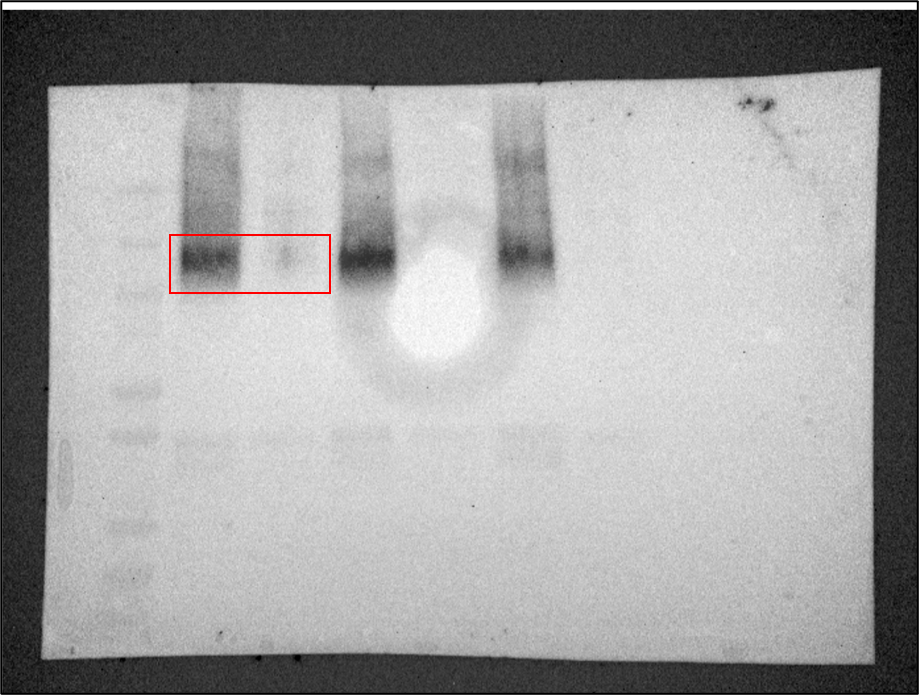

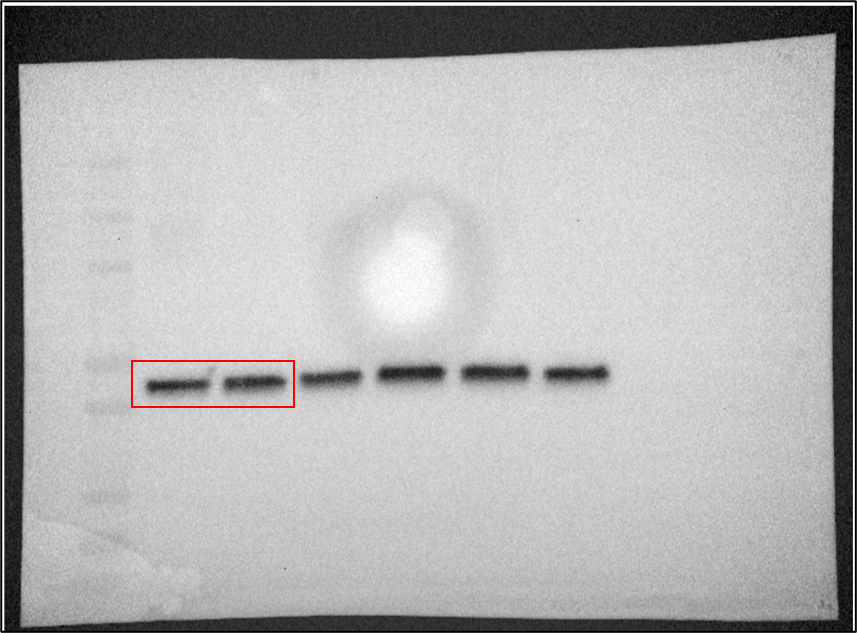


Supplementary Figure 1D: Top: MCM2, bottom: β-actin


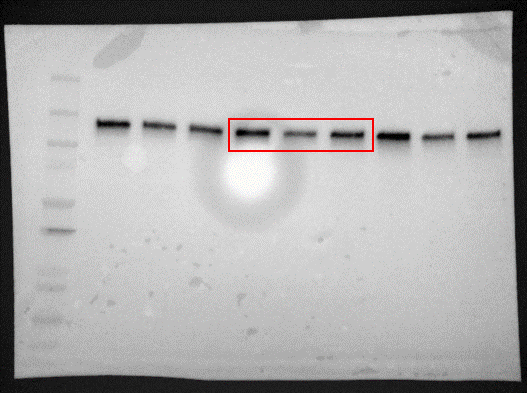

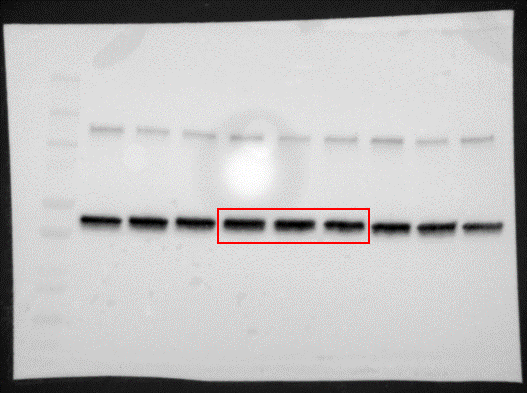


Figure 4: MCM6 (top band) and β-actin (bottom band)


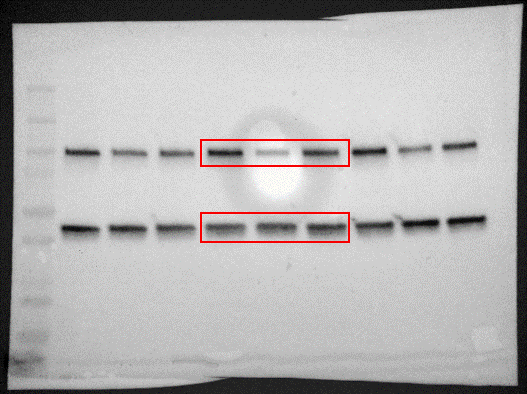


Supplementary Figure 1F: Top: MCM7, bottom: β-actin


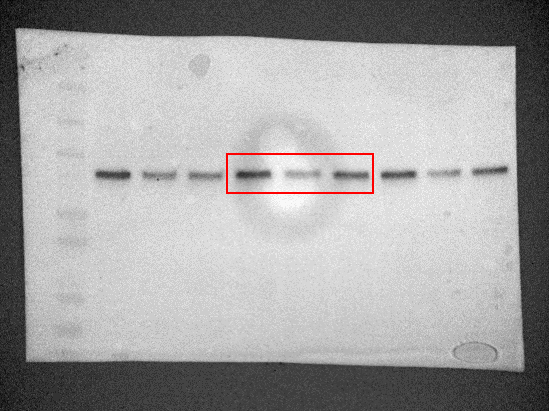

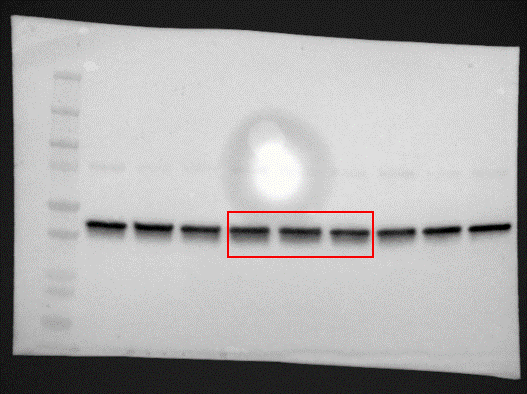

Supplement: Supplementary file 1 — Supplementary Fig. 1 [file mmc1.docx]
